# The Interaction of Heptakis (2,6-di-O-Methyl)-β-cyclodextrin with Mianserin Hydrochloride and Its Influence on the Drug Toxicity

**DOI:** 10.3390/ijms22179419

**Published:** 2021-08-30

**Authors:** Sylwia Belica-Pacha, Magdalena Małecka, Mateusz Daśko, Katarzyna Miłowska, Maria Bryszewska, Grażyna Budryn, Joanna Oracz, Bartłomiej Pałecz

**Affiliations:** 1 Unit of Biophysical Chemistry, Department of Physical Chemistry, Faculty of Chemistry, University of Lodz, Pomorska 165, 90-236 Lodz, Poland; magdalena.malecka@chemia.uni.lodz.pl (M.M.); bartlomiej.palecz@chemia.uni.lodz.pl (B.P.); 2 Department of Inorganic Chemistry, Faculty of Chemistry, Gdansk University of Technology, Narutowicza 11/12, 80-233 Gdansk, Poland; mateusz.dasko@pg.edu.pl; 3 Department of General Biophysics, Faculty of Biology and Environmental Protection, University of Lodz, Pomorska 141/143, 90-236 Lodz, Poland; katarzyna.milowska@biol.uni.lodz.pl (K.M.); maria.bryszewska@biol.uni.lodz.pl (M.B.); 4 Institute of Food Technology and Analysis, Faculty of Biotechnology and Food Sciences, Lodz University of Technology, Stefanowskiego 4-10, 90-924 Lodz, Poland; grazyna.budryn@p.lodz.pl (G.B.); joanna.oracz@p.lodz.pl (J.O.)

**Keywords:** mianserin hydrochloride, heptakis (2,6-di-O-methyl)-β-cyclodextrin, isothermal titration calorimetry, circular dichroism, mass spectrometry, molecular docking, cytotoxicity

## Abstract

One tetracyclic antidepressant, mianserin hydrochloride (MIA), has quite significant side effects on a patients’ health. Cyclodextrins, which are most commonly used to reduce the undesirable features of contained drugs within their hydrophobic interior, also have the potential to alter the toxic behavior of the drug. The present paper contains investigations and the characteristics of interaction mechanisms for MIA and the heptakis (2,6-di-O-methyl)-β-cyclodextrin (DM-β-CD) system, and evaluated the effects of the complexation on MIA cytotoxicity. In order to assess whether there was an interaction between MIA and DM-β-CD molecules, isothermal titration calorimetry (ITC) have been chosen. Electrospray ionization mass spectrometry (ESI-MS) helped to establish the complex stoichiometry, and circular dichroism spectroscopy was used to describe the process of complex formation. In order to make a wider interpretative perspective, the molecular docking results have been performed. The viability of Chinese hamster cells were investigated in the presence of DM-β-CD and its complexes with MIA in order to estimate the cytotoxicity of the drug and the conjugate with the chosen cyclodextrin. The viability of B14 cells treated with MIA+DM-β-CD is lower (the toxicity is higher) than with MIA alone, and no protective effects have been observed for complexes of MIA with DM-β-CD in any ratio.

## 1. Introduction

**Figure 1:**
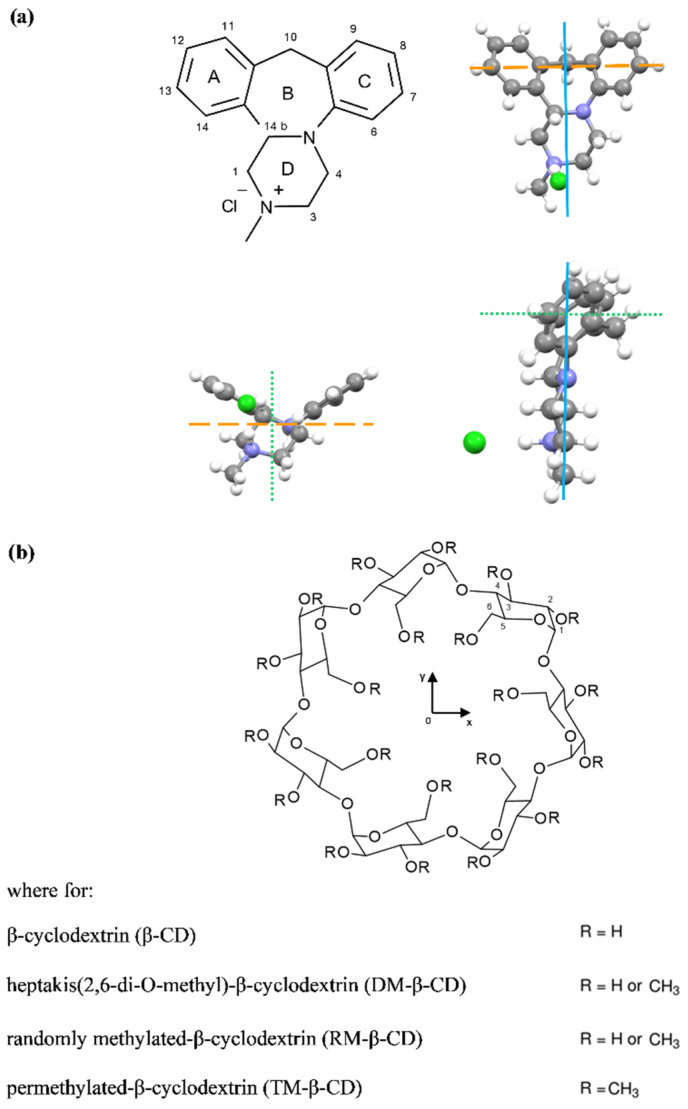
Structures of (**a**) mianserin hydrochloride and (**b**) β-cyclodextrins.

Antidepressants were prescribed very often before SARS-CoV-2 [1], and during the COVID-19 pandemic, the usage increased [2]. Mianserin (MIA) is a noradrenergic and specific serotonergic antidepressant [3] that is mostly used as hydrochloride salt [4] (Figure 1a).

Mianserin also has additional beneficial properties [3], like for example the ability to stabilize blood sugar levels [5], or as a candidate to depleting ergosterol levels in the pathogen visceral Leishmaniasis [6]. However, the prolonged use of psychotropic drugs could cause side effects such as hepatotoxicity [7,8] or cardiotoxicity [9]. During the previous work [10], we have attempted to ameliorate the pharmaceutical features of mianserin hydrochloride by inclusion inside β-cyclodextrin molecules. We found that the MIA–β-CD complex decreased drug toxicity [10]. In the present study, we wanted to check whether the methylated derivative of β-cyclodextrin—i.e., heptakis (2,6-di-O-methyl)-β-cyclodextrin, which has greater water solubility—will have the same effect. Moreover, in the near future, we are going to test other β-cyclodextrin derivatives, both non-ionic (e.g., 2-hydroxypropyl-β-cyclodextrin) and ionic (e.g., carboxymethyl-β-cyclodextrin sodium salt) as well. Cyclodextrins (Figure 1b) are mostly used in order to improve the solubility of drugs in water [11,12]. They are supramolecules with a hydrophobic interior, which may include hydrophobic parts of the drug molecule and hydrophilic shells, consisting of primary and secondary hydroxyl groups that can interact with water molecules [13,14]. In pharmacological tests of the mianserin antidepressant effect [15], higher potency is found for the optical enantiomer (S)-(+)-mianserin. However, we chose the racemate for testing, since commercially available mianserin hydrochloride is a racemic mixture. In this work, we mostly examined the physicochemical properties of mianserin hydrochloride during interaction with heptakis (2,6-di-O-methyl)-β-cyclodextrin (DM-β-CD) by an isothermal titration calorimetry (ITC), electrospray ionization mass spectrometry (ESI-MS), and circular dichroism spectroscopy (CD) to see how the properties of the drug may change when methyl groups appear in native β-CD. First of all, we chose DM-β-CD mainly because of the known methylation positions (2 and 6); however, we intend to investigate other β-cyclodextrin derivatives in order to compare and potentially find possible relationships, for example the enthalpy–entropy compensation. Moreover, to make a wider interpretative perspective of the studied systems, molecular docking (MD) simulations have been carried out not only for DM-β-CD, but also for the other methylated β-CD and the native β-CD. Most importantly, the effects of DM-β-CD and its complexes with MIA on the viability of Chinese hamster cells were investigated. On the basis of the obtained results, we try to estimate the cytotoxicity of the drug in the presence of methylated β-cyclodextrin. As it turned out, the viability of B14 cells treated with MIA+DM-β-CD was lower than with MIA alone, which was the opposite effect to that observed for native β-cyclodextrin [10], where Chinese hamster cells’ viability was protected and MIA toxicity was reduced in the β-CD presence. Once again, no protective effect has been observed for complexes of MIA with DM-β-CD in any ratio. Thus, the MIA+DM-β-CD should rather be used in situations where the toxicity gain is desirable, e. g., in anti-tumor cell potential or Leishmaniasis treatment.

## 2. Results and Discussion

### 2.1. Isothermal Titration Calorimetry (ITC)

**Figure 2:**
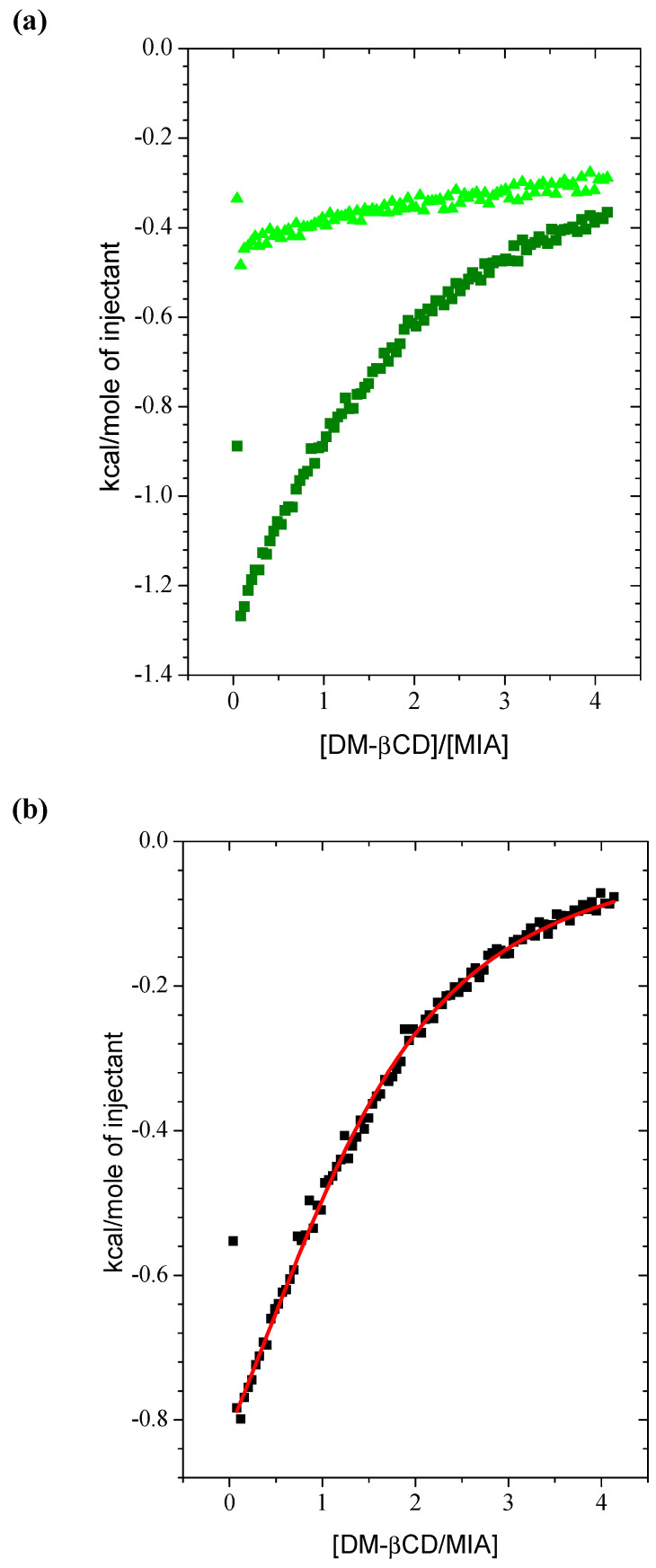
(**a**) The titration integrated thermal effects of administration of a 15 mM DM-β-CD solution (in a syringe) to a 0.79 mM MIA solution (in a cell) at 298.15 K under atmospheric pressure *p* = 101,000 Pa (■) and dilution of 15 mM DM-β-CD solution in water (▲); (**b**) the integrated heat of interaction between MIA and DM-βCD molecules (■) corrected by the heat of the correspondent dilution. The solid red line represents the best non-linear least squares fit to a mathematical model of “One set of binding sides” [17,23].

In order to assess whether there was an interaction between mianserin hydrochloride and heptakis (2,6-di-O-methyl)-β-cyclodextrin molecules, isothermal titration calorimetry (ITC) has been chosen. The ITC method permits the simultaneous determination of the equilibrium constant (K), enthalpy (ΔH), and stoichiometry coefficient of binding (n) from one titration set of experimental data using the least squares non-linear fit based on the Wiseman isotherm (Figure 2; Table 1) [16]. Moreover, the entropic effect (TΔS) together with the Gibbs free energy value (ΔG) were calculated from them (Table 1). A mathematical model called “One-sets of sites” [17,18] was used, selected from the options implemented in the ORIGIN 7 program. This model has been chosen because of the smallest errors of the fitted parameters [19] in comparison to the “Two-sets of sites model” [19,20] or the “Sequential model” [20,21]. The association constant value for MIA and DM-β-CD molecules represents a moderate strength of interaction, since it is greater than 10 M^−1^ and less than 10^7^ M^−1^ [17]. The interaction between MIA and DM-β-CD is a little bit stronger than that between MIA and the native β-CD molecule, as indicated by the values of the equilibrium constant and the absolute value of Gibbs free energy of binding compiled for both complexes (Table 1). However, the similar comparison made for systems with other antidepressant drug-sertraline hydrochlorides and the same β-cyclodextrins from the literature [22] (Table 1) gives information that the discrepancies of K or ΔG for MIA interacting with two different β-CDs are rather small. In other words, the methyl groups on the DM-β-CD molecule helped the mianserin hydrochloride molecule to enter the DM-β-CD cavity to a lesser extent than in the case of the sertraline hydrochloride molecule (Table 1) [22].

The complexation process during interactions of MIA with DM-β-CD runs spontaneously, as indicated by a negative value of the Gibbs free energy (Table 1). Furthermore, the effect of interaction between MIA and DM-β-CD is exothermic (ΔH < 0), but the enthalpic effect of binding for the discussed molecules has been dominated by the entropic effect of complex formation (|ΔH| < |TΔS|).

This made it clear that the entropy increment occurring during inclusion due to desolvation (the release of water molecules that were originally installed in the cavity of the cyclodextrin and the desolvation of peripheral hydroxyl or substituted hydroxyl groups as well as the guest molecules [24]) is quite meaningful (Table 1).

Thus, the complexation is driven both by the enthalpy and the entropy change, but the entropic term controls the complex’s ultimate stability. The described relation of enthalpic and entropic effect values to each other presents the opposite situation than the case from the literature for sertraline hydrochloride and the same β-cyclodextrins (Table 1), where the enthalpic effects have greater absolute values than the entropic ones.

The binding constant of MIA inclusion inside the DM-β-CD cavity is higher than for the native β-CD (Table 1). This is probably due to a better fitting of the mianserin hydrochloride molecule into the more apolar DM-β-CD cavity, but not so successfully as the sertraline hydrochloride molecule, which has an almost five times greater value of binding constant (Table 1).

The stoichiometry coefficient value of the complex formation between DM-β-CD and MIA molecules placed near to the ratio of 1.5, while the coefficient value for the native β-CD is almost equal to the value of 2 (Table 1). This may suggest that some part of MIA molecules could interact with one DM-β-CD molecule, and some other MIA molecules simultaneously interact with two DM-β-CD molecules, but the fraction of these latest connections is not as high as in the complex formation between MIA and β-CD molecules (Table 1).

### 2.2. ESI-MS/MS Analysis

**Figure 3:**
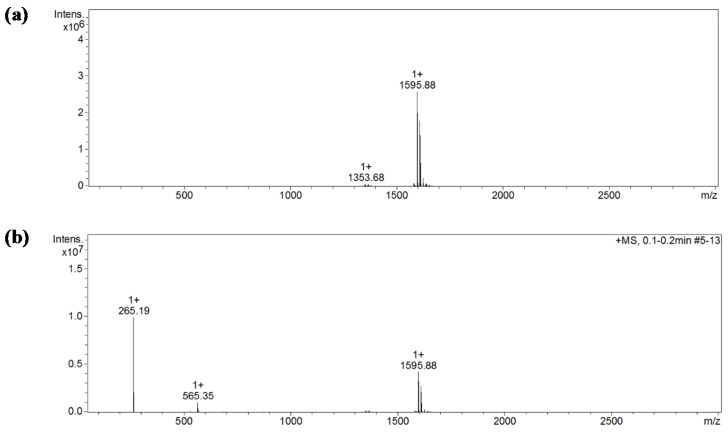
The spectrum of MIA+DM-β-CD: (**a**) The range of scanning from 100 to 3000 *m/z*. MS mode; MS/MS spectra in the MRM mode, without fragmentation-CID energy 0 eV, with mianserin + DM-β-CD at 1595.88 *m/z,* and probably some amount of RM-β-CD at 1353.68 *m/z* as impurity with degree of substitution (DS) between 15 and 16. This is higher than 14, which is an average DS characteristic for DM-β-CD. (**b**) The range of scanning from 100 to 3000 *m/z*. MS mode; MS/MS spectra in the MRM mode, with fragmentation-CID energy 50 eV, with mianserin at *m/z* 265.19, mianserin + DM-β-CD at 1595.88 *m/z,* and dimer mianserin + mianserin hydrochloride at *m/z* 565.35.

Electrospray ionization mass spectrometry (ESI-MS) provides a powerful tool to study non-covalent “host-guest” inclusion complexes of cyclodextrin and hydrophobic groups of molecules, or whole hydrophobic molecules and the mild ionization procedure, which allows the structure of examined complexes to be maintained [25]. Identification of the complex formation between heptakis (2,6-di-O-methyl)-β-cyclodextrin molecules and mianserin hydrochloride was performed with the use of the ESI-MS/MS technique (Figure 3). The basic spectrum (Figure 3a) showed the most intense peak at *m/z* 1595.88, which pointed out the predominant presence of the mianserin and DM-β-CD combination in the molecular ratio 1:1 [MIA+DM-β-CD+H]^+^. In a much smaller amount, at *m/z* 1353.68, came the β-CD derivative with hydroxyl groups with a substitution degree higher than 14, as it should be for DM-β-CD themselves (Figure 3a). Moreover, except for the already registered peak at *m/z* 1595.88, fragment ions at *m/z* 265.19 for voltage of 20 eV ionization have been produced (Figure 3b), corresponding with the existence of a mianserin molecule [26]. The spectrum additionally showed a peak at *m/z* 565.35, which is very likely for a dimer of mianserin + mianserin hydrochloride [MIA_2_+Cl+H]^+^. It is also worth emphasizing that in the described MIA+DM-β-CD case, the peak for the 1:2 connection (which was registered in the complex of MIA+β-CD [10]) was not detected, thus possibly indicating that connections with higher stoichiometry are less stable under the measurement conditions of this technique, such as a primarily high temperature.

### 2.3. Circular Dichroism (CD) Spectroscopy

**Figure 4:**
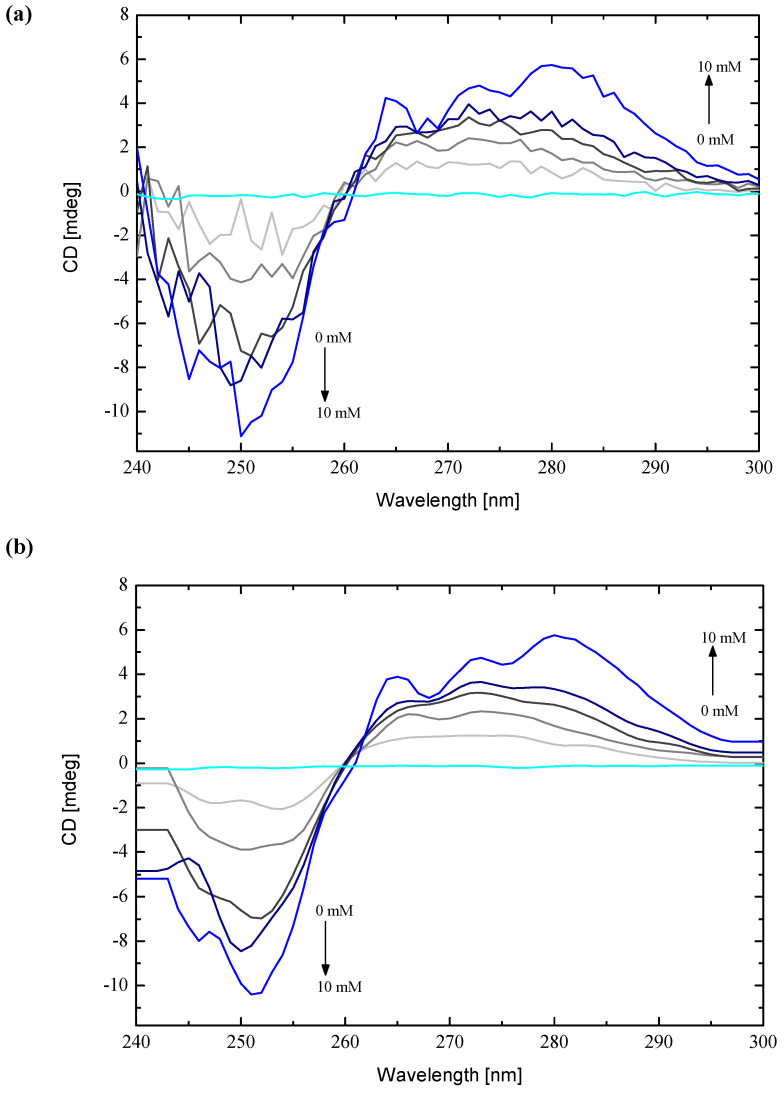
Selected circular dichroism spectra for aqueous solutions of mianserin hydrochloride with constant 0.6 mM concentration, together with growing content of heptakis (2,6-di-O-methyl)-β-cyclodextrin from 0.3 mM to 10 mM, with stoichiometry from 0.5:1 (light grey) to 1:16 MIA–DM-β-CD (blue line), and spectra for free DM-β-CD (cyan line). (**a**) Raw data and (**b**) smoothed with Savitzky–Golay filter [33,34] implemented as one of the smoothing options in the ORIGIN v. 7.0 software [35].

In an aqueous environment, mianserin hydrochloride did not present any crucial signals in the range of 240–300 nm at the circular dichroism spectrum [10]. Heptakis (2,6-di-O-methyl)-β-cyclodextrin also revealed slightly visible features during registration of the circular dichroism spectrum (Figure 4). The intermixture of MIA and DM-β-CD molecules in water produced a connection, which gave an induced signal during circular dichroism measurements (ICD) [27]. The ICD spectra for the MIA+DM-β-CD system—which consists from the unchanging content of MIA and the constantly increasing content of DM-β-CD—are quite noisy, especially in the region from 240 to 260 nm (Figure 4a), for any quantitative analysis.

However, with increasing DM-β-CD content, there can be observed a fairly strong negative Cotton effect for the main 250 nm absorption band, along with an almost twofold weaker positive effect, changing the positions of maximum from around 260 nm and 270 nm to 280 nm of shoulder band for the case of MIA with the most concentrated solution of DM-β-CD (Figure 4). As it has been stated in the literature [28,29,30,31], the sign and the intensity of the ICD signals of cyclodextrin complexes are structure-dependent, and can be used to identify possible arrangements of the guests in relation to the host. According to Kodaka [28,29] and Harata [32], a positive Cotton effect is related to the parallel orientation of the electric transition dipole moment of a chromophore located in the cavity of cyclodextrin with respect to the Z-axis of the macromolecule cavity. On the other hand, a negative Cotton effect is related to the perpendicular orientation. This point of view seems to agree very well with the obtained results, namely the observation of the negative and the positive Cotton effect (Figure 4) for the MIA molecule during inclusion to the DM-β-CD cavity. This phenomenon can be explained by the fact that the MIA molecule has at the same time the parallel and perpendicular orientation of the electric transition dipole moment of chromophores to the Z-axis of the DM-β-CD cavity, because it has four rings forming a shape similar to a bent letter T (Figure 1a), with the chromophores almost perpendicular to each other. The intensity of the ICD signal may be connected with the deepness of inclusion inside the DM-β-CD cavity of the MIA molecule [31].

Based on this, one can assume that the A-ring and the B-ring of the MIA molecule are probably deeply inserted into the cavity of DM-β-CD with the dipole moment of chromophores (aromatic benzene rings) perpendicular to the Z-axis of DM-β-CD (Figure 1) for which the negative signal with a wavelength of 250 nm corresponds (Figure 4).

As some authors stated, the parallel-polarized electronic transition along the axis of the cavity should give an ICD signal that is two times larger, with the opposite sign as compared with the ICD signal caused by a perpendicular-polarized one [29,31]; however, the presented results showed the reversed situation, where there were two times more intense negative ICD signals than positive ones (Figure 4). Moreover, the inverted sign of the ICD signal has been observed [29,30] when the chromophore was located partially outside the cyclodextrin, or when the guest molecule was moved from the inside to the outside of the cavity. If such a situation has happened in the examined MIA+DM-β-CD system, then the bands with opposite signs originate from one part of the MIA lying inside the cavity of the DM-β-CD, and the other one outside the cavity. The next part of the paper will explore which of the two explanations is more possible, using a molecular docking experimental results latter.

Furthermore, when the results of ICD obtained for the MIA interacting with plain β-CD [10] are compared with the presented outcomes, the decrease in positive signal intensity for the MIA complex with DM-β-CD is observed. It is possibly coming from the minimal increase in the cavity size, resulting in a slightly worse fit [31]. However, the negative Cotton effect for MIA inside DM-β-CD is much more minus than the negative signal of the MIA+β-CD connection. This could be attributed to the different positions of the MIA molecule inside both cyclodextrins, which emphasizes the considerable differences of these two macromolecules.

### 2.4. Molecular Docking

**Figure 5:**
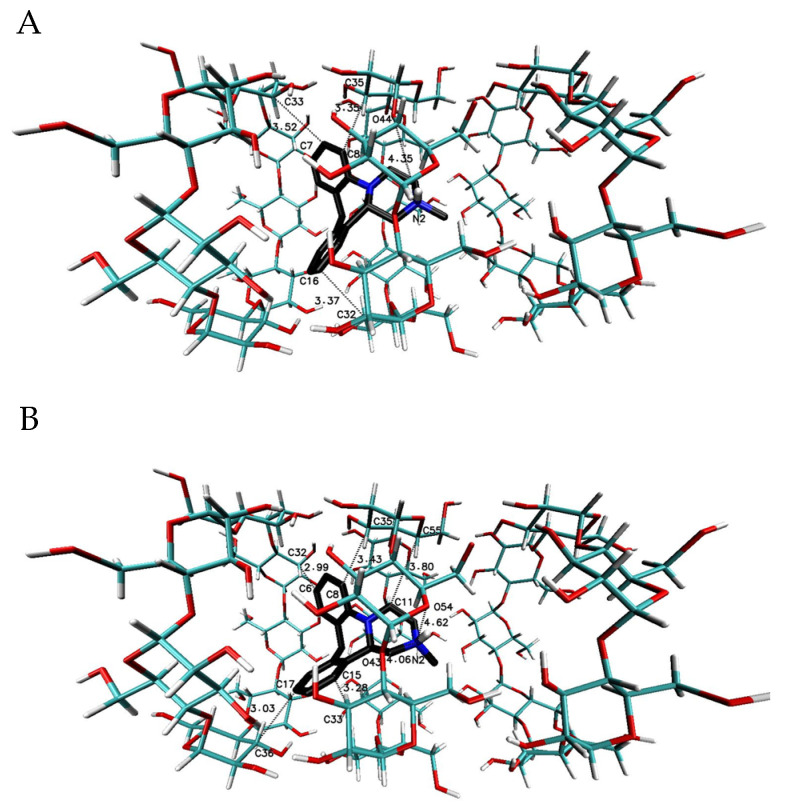
The chosen geometries of the MIA–β-CD complex with stoichiometry 1:3 (I-II-III or “head-to-head-to-tail” orientation of β-CD molecules to each other [36,37]) obtained by the Molecular Docking (MD) simulations, with the use of β-CD crystal structure with refcode 648855 from The Cambridge Structural Database (CSD) [38]; (**A**) for S-(+)-MIA·HCl and (**B**) for R-(−)-MIA·HCl.

**Figure 6:**
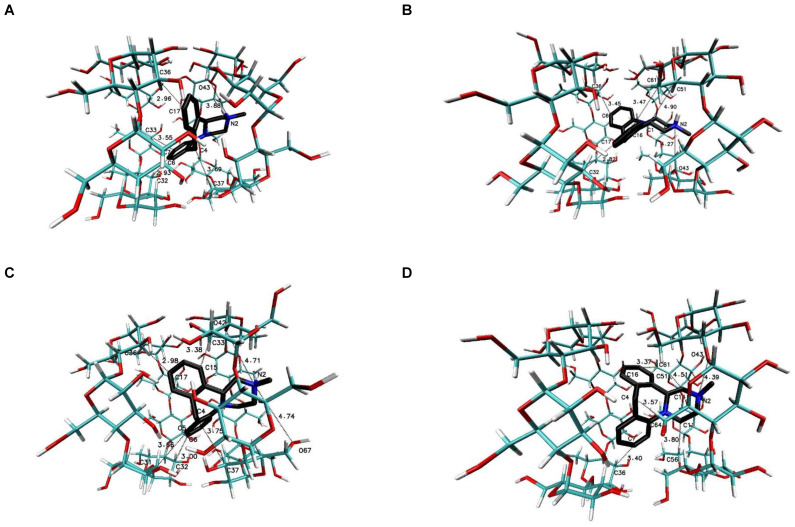
The chosen geometries of the MIA–β-CD complex with stoichiometry 1:2 (I-II or “head-to-head” orientation of β-CDs for (**A**,**C**), and II-III, or “head-to-tail” orientation of β-CDs molecules to each other [36,37] for (**B**,**D**) obtained by MD simulations with the use of β-CD crystal structure with refcode 648855 from the CSD data base [38]; (**A**,**B**) for S-(+)-MIA·HCl and (**C**,**D**) for R-(−)-MIA·HCl.

**Figure 7:**
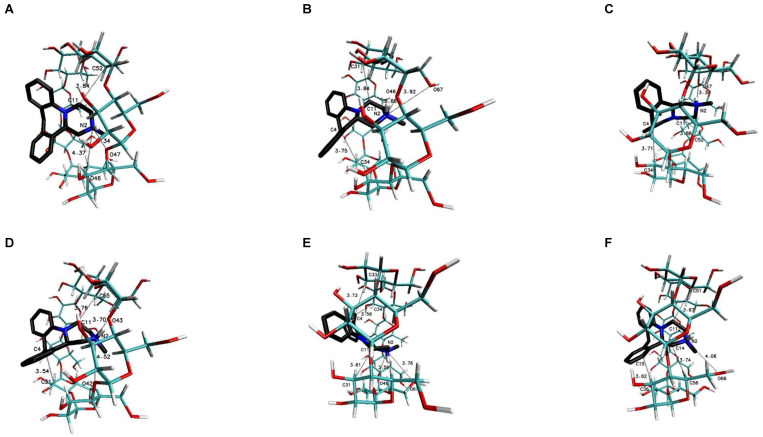
The chosen geometries of the MIA–β-CD complex with stoichiometry 1:1. I for (**A**,**D**); II for (**B**,**E**); and III for (**C**,**F**) obtained by MD simulations with the use of β-CD crystal structure with refcode 648855 from the CSD data base [38]; (**A**–**C)** for S-(+)-MIA·HCl and (**D**–**F**) for R-(−)-MIA·HCl.

**Figure 8:**
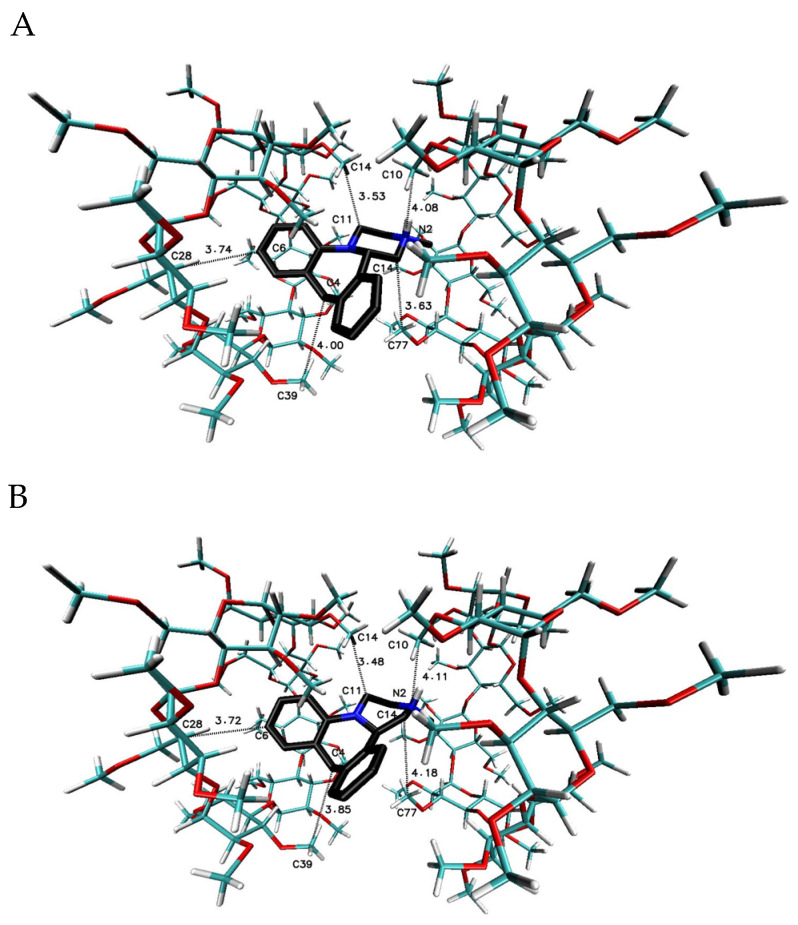
The chosen geometries of the MIA–TMβ-CD complex with stoichiometry 1:2 (I-II or “head-to-head”) obtained by MD simulations with the use of TMβ-CD crystal structure with refcode ALIGAE from the CSD data base [39]; (**A**) for S-(+)-MIA·HCl and (**B**) for R-(−)-MIA·HCl.

**Figure 9:**
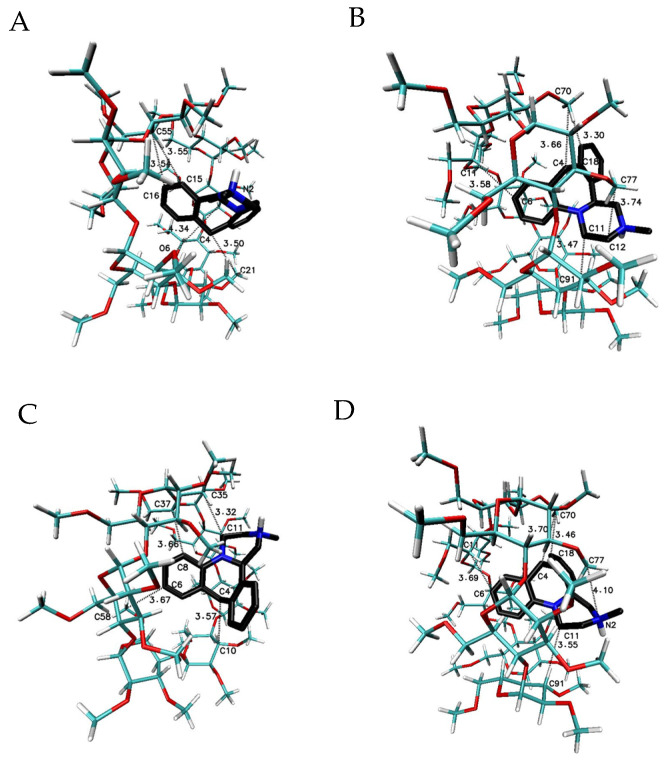
The chosen geometries of the MIA–TMβ-CD complex with stoichiometry 1:1 (I for (**A**,**C**) and II for (**B**,**D**) obtained by MD simulations with the use of TMβ-CD crystal structure with refcode ALIGAE from the CSD data base [39]; (**A**,**B**) for S-(+)-MIA·HCl and (**C**,**D**) for R-(−)-MIA·HCl.

**Figure 10:**
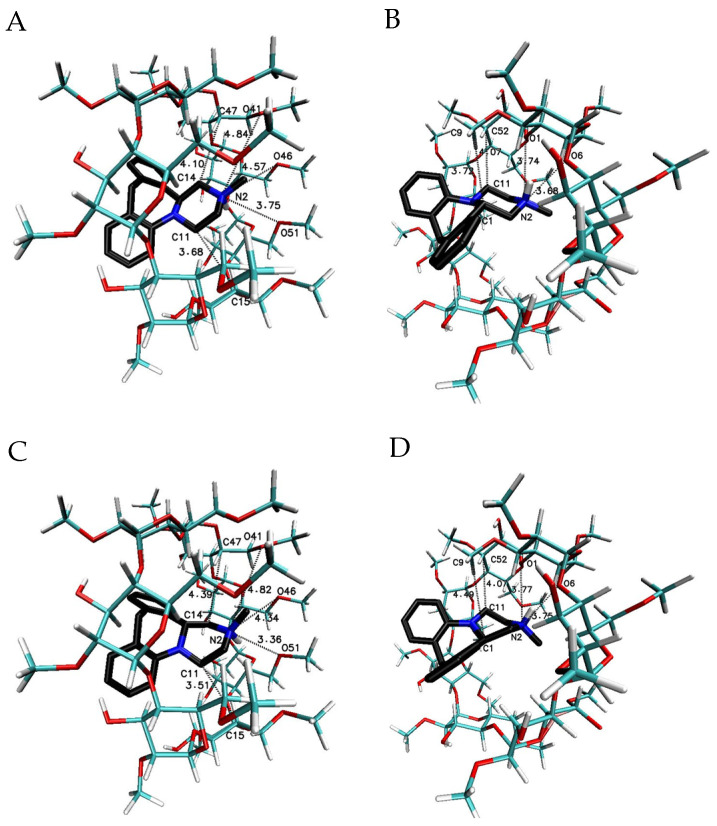
The chosen geometry of the MIA–DMβ-CD and MIA–RMβ-CD complexes with stoichiometry 1:1 obtained by MD simulations with the use of DMβ-CD or RMβ-CD crystal structure with refcode BEFJOL for DMβ-CD and JOSWOD for RMβ-CD from the CSD data base [40,41]; (**A**) or (**B**) for S-(+)-MIA·HCl and (**C**) or (**D**) for R-(−)-MIA·HCl.

In order to make a wider interpretative perspective of molecular docking results, the MIA molecule has been examined with native β-CD and other β-CD methylated derivatives (Table 2; Figure 5, Figure 6, Figure 7, Figure 8, Figure 9 and Figure 10). The obtained free energy of binding values for all considered complexes fall within a rather narrow range, from −5.9 to −8.5 kcal∙mol^−1^, which means that the differences in the magnitude of the energies are noticeable, but not very large. Moreover, more optional energies have been received for the MIA molecule connected with more than one cyclodextrin host molecule—both native or methylated—in comparison to 1:1 complexes (Table 2). The highest stoichiometry of the studied complexes depended on the number of selected cyclodextrin molecules in the crystal cell available in the Cambridge Structural Database (CSD) (Table 2).

The most favorable free energy of binding has been calculated for the complex with the MIA–β-CD ratio equal to 1:3, except for the R-(−)-MIA·HCl enantiomer (Table 2). In Figure 5, the examples of the MIA-β-CD complex geometries for both enantiomers with stoichiometry 1:3 obtained by MD simulations have been placed. As it can be seen (Figure 5), the guest molecule of mianserin interacts primarily with the center β-CD molecule, and the complex is stabilized by two other host molecules. After the removal of one of the peripheral β-CD molecules and re-optimization of the binding free energy for the MIA+2βCD aggregate (Table 2; Figure 6), the obtained energy values have been raised by about 0.4 kcal∙mol^−1^ for the MIA included inside of the two β-CDs oriented “head-to head” to each other, and by 0.9 kcal∙mol^−1^ for the “head-to-tail” orientation of the β-CDs (Table 2). From our previous quantum chemical calculation research [14], it appears that the “head-to-head” orientation of two β-CDs—with or without the MIA molecule inside—is energetically beneficial, mostly because of the hydrogen bonds formation between hydroxyl groups on the secondary carbon atoms in β-cyclodextrins. This hydrogen bonds formation has a major impact on the computed complexation energies [14]. It is possible that this is the reason why the free energies of binding (for both enantiomers of MIA; Table 2) changed a little bit more rapidly after the removal (from the original MIA+3βCD aggregate) of one β-CD molecule for “head-to-tail” β-CD orientation than for “head-to-head” during the molecular docking examinations. For three 1:1 MIA–β-CD, results the energies are slightly less favorable than for higher ratios of binding, and are very similar to each other regardless of the type of the docked MIA enantiomer (Table 2). Two molecules of TMβ-CD create more stable connections with the MIA molecule in comparison to “head-to-tail” β-CD orientation and 1:1 complexes of the rest methylated β-CDs (Table 2). As it is shown in Figure 5, Figure 6, Figure 7, Figure 8, Figure 9 and Figure 10, for the case of three β-CDs, the whole MIA molecule is set inside of them (Figure 5), and the same situations occur for 1:2 complexes for β-CD and its methylated derivative TMβ-CD (Figure 6 and Figure 8).

When the 1:1 complexes are analyzed, the more or less deep but partial insertion of the MIA molecule in the macrocycle cavity is observed for all presented cyclodextrins (Figure 7, Figure 9 and Figure 10).

### 2.5. Cytotoxicity

**Figure 11:**
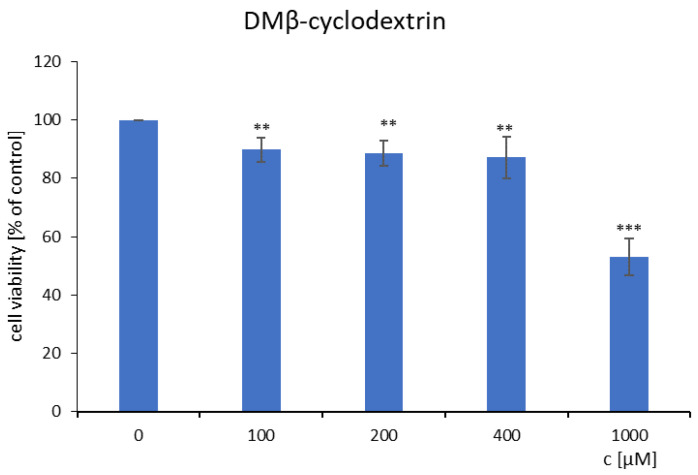
The Chinese hamster cells (B14) viability incubated with heptakis (2,6-di-O-methyl)-β-cyclodextrin after 24 h. Error bars represent S.D. ** *p* < 0.01 *** *p* < 0.001 confronted with the untreated cells.

**Figure 12:**
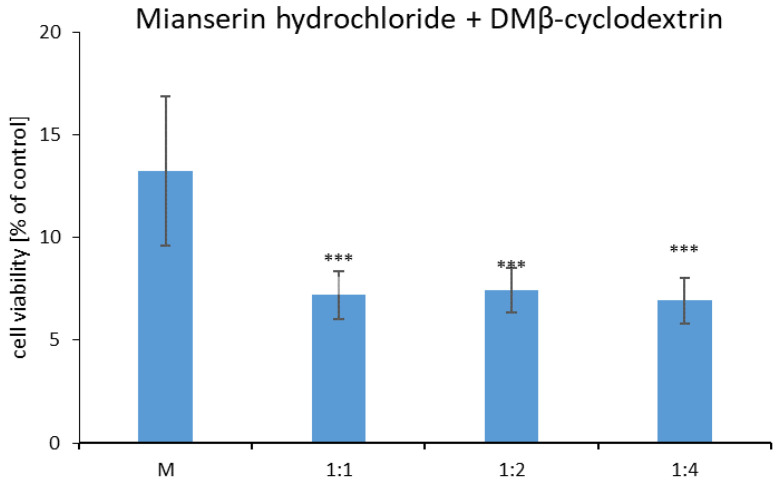
The Chinese hamster cells (B14) viability incubated with mianserin hydrochloride-heptakis (2,6-di-O-methyl)-β-cyclodextrin mixture after 24 h. Error bars represent S.D. *** *p* < 0.001 versus cells incubated with 200 µM mianserin hydrochloride solution [10].

The viability of Chinese hamster cells (B14) was investigated in the presence of heptakis (2,6-di-O-methyl)-β-cyclodextrin alone and together with mianserin hydrochloride (Figure 11 and Figure 12). The toxicity of MIA alone has been measured previously [10], and based on these results, the appropriate concentration of MIA was selected. The joint action of MIA and DM-β-CD compounds were examined by the MTT spectrophotometric method. The outcomes were shown in relation to the untreated cells (the control), which were taken as one hundred percent. The cell viability decreased for all concentrations of mianserin hydrochloride (10–1000 μM) [10]. For further investigations with DM-β-CD, the concentrations 200 µM of mianserin hydrochloride was selected, because the cell viability treated by the MIA decreased to about 15% of the control (Figure 12). After 24 h exposure to heptakis (2,6-di-O-methyl)-β-cyclodextrin alone, the average reduction in the percentage of viable cells was observed (Figure 11). The B14 cells viability cultured with the DM-β-CD were mostly above 80 percent, except for the concentration of 1000 µM, which was considered to be toxic. At the concentration of 1000 µM of DM-β-CD, the viability of B14 cells decreased to 53% in relation to the control (Figure 11). This methylated derivative of β-cyclodextrin under 400 µM is still not toxic (Figure 11), and we decided to choose that concentration to investigate whether DM-β-CD can protect Chinese hamster cells from the toxic effects of mianserin hydrochloride, or make the drug more toxic. In the literature [42,43,44], the enclosing of the drug by native β-cyclodextrin macromolecules increased the cytotoxicity against examined tumor cell lines. A similar situation has been observed for DM-β-CD+MIA complexes—but in the presented case, the cell viability of Chinese hamster non-tumor cells incubated with the complexes was remarkably lower when confronted with the viability cells treated only with the 200 μM MIA solution (Figure 12). The quantity of the cells in the presence of the complexes was equal only to about 6–7% of the control, and the toxic effect was profound. The same was found for all complexes used independently from the MIA–DM-β-CD molar ratio (Figure 12).

## 3. Materials and Methods

### 3.1. Materials

Mianserin hydrochloride (MIA), heptakis (2,6-di-O-methyl)-β-cyclodextrin (DM-β-CD), methyl-thiazolyl-diphenyl-tetrazolium bromide (MTT), phosphate buffered saline (PBS) tablets, and fetal bovine serum were obtained from Sigma-Aldrich. The grade of purity for the mentioned substances were placed in Table 3. A high grade of purity water (LC-MS CHROMASOLV^®^) was used in the ESI-MS measurements. For the ITC and the CD experiments, three times distilled and degassed water was used.

### 3.2. Methods

#### 3.2.1. Process of MIA–DM-β-CD Aggregates Formation

The MIA and DM-β-CD molecules formed aggregates in water at once during mixing, which was confirmed by the results from the ITC measurements. Before the procedure of complex formation, the drug and cyclodextrin were dried and weighed separately with the use of a Binder dryer [45] and a Mettler AE240 analytical balance [10]. For ESI-MS measurements, the DM-β-CD and MIA were dissolved in LC-MS CHROMASOLV^®^ water. For MTT assay, the PBS water solution (pH 7.4) was used. Three-times distilled and degassed water was applied for circular dichroism spectroscopy. After easy and fast solubilization of MIA and DM-β-CD, the stock solutions were stirred together for 15 min to obtain an MIA–DM-β-CD mixture with proper molar ratios for appropriate experiments [10].

#### 3.2.2. Isothermal Titration Calorimetry (ITC)

The thermodynamic parameters of interaction between mianserin hydrochloride and heptakis (2,6-di-O-methyl)-β-cyclodextrin molecules were determined during isothermal calorimetric titration carried out on the VP-ITC device from MicroCal (USA) at 298.15 K (pH 6.80). The measuring cell with a volume of 1.4275 mL was filled with an aqueous 0.80 mM solution of MIA. A solution of 15 mM DM-β-CD [17] was added into the measuring cell by injecting 94 portions of 3 µL with 600 s intervals—which was sufficiently long for the signal to return to the baseline—and a stirrer rotational speed of 264 rpm. It is a very common situation that, although cyclodextrin is a macromolecule, it is used as a titrant, and the drug/ligand is placed in the measuring cell [17,46,47], mostly because of the stoichiometry drug to cyclodextrin being different from 1:1 and higher for cyclodextrin [14,46,48]. From a mathematical point of view, the procedure of parameter calculations has been not changed, as if the macromolecule (in the presented case, the drug molecule played such a role) were in the cell. The direct energetical effects of interaction between MIA and DM-β-CD molecules in an aqueous solution were obtained from the main titration experiment, corrected by the dilution effects of the DM-β-CD solution in pure water. The dilution effects of the MIA solution by pure water were small enough to be omitted. The subsidiary measurements were carried out with the use of the same procedure and the concentrations of reagents, as in the case of the main experiment. The example of interaction between MIA and DM-β-CD molecules and dilution effects together with corrected result were placed on Figure 2a,b. The integrated heat of interaction was analyzed as a function of the DM-β-CD/MIA ratio (Figure 2a,b). The first point from the first injection (3.0 μL) was discarded—considering the solution diffusion effects between the syringe and the calorimetric cell [47]—and the data were fitted from the second one by a non-linear least squares method using the ORIGIN v. 7.0 software [35] supplied with the calorimeter. The calculated parameters were obtained as the average values from the four independent experiments, and the results were gathered in Table 1.

#### 3.2.3. ESI-MS/MS Analysis

The substances were examined with the use of the positive ESI-MS/MS mode [25] on an ultrahigh resolution hybrid quadrupole/time of-flight mass spectrometer (UHR-Q-TOF-MS/MS, maXis™, Bruker, Bremen, Germany). Solutions of mianserin hydrochloride together with heptakis (2,6-di-O-methyl)-β-cyclodextrin in water with the twofold excess of DM-β-CD in comparison to MIA were transferred into the electrospray ion source (ESI), with a flow rate of 100 µL/min through a syringe pump. The complexes were examined with the use of a multiple reaction monitoring mode (MRM) during registration with a scan range from 100 to 3000 *m/z* of the MS/MS spectra. The nitrogen (temperature 593 K and pressure 1 bar) was the nebulizer and the collision gas with a flow rate of 8.0 L/h. The MS/MS spectra were obtained in collision-induced dissociation (CID) mode using nitrogen as the collision gas. In the MS/MS measurements, the energies ranges were from 0 to 50 eV for each MRM transition for the dissociation of samples. There were recorded and characterized signals with a positively charged molecular ion ([M + H]^+^). Instrument control, data acquisition, and evaluation were done with the OTOFControl 3.2 and HyStar 3.2 software, respectively.

#### 3.2.4. Circular Dichroism (CD) Spectroscopy

The circular dichroism experiments were carried out for 0.6 mM mianserin hydrochloride solutions, together with changing heptakis (2,6-di-O-methyl)-β-cyclodextrin content from 0.1 to 10 mM with the use of a Jasco J-815CD spectropolarimeter (Japan) at 298.15 K. The spectra were registered from 240 nm to 300 nm in 10-mm path length Helma quartz cuvettes with a scan rate of 50 nm/min (as well as a response time of 4 s and a wavelength step of 1 nm). The result was an average calculated from three acquisitions. Through the CD spectropolarimeter, the nitrogen was passed to cool and remove oxygen in order to avert ozone production. A water blank sample was recorded to compensate for the baseline drift in the CD spectra.

#### 3.2.5. Computational Studies

##### Ligands and Macromolecule Preparation for Molecular Docking

The 3D structures of S-(+)-MIA·HCl were prepared with the use of a crystal structure, with a refcode of HIJDEJ [49] from Cambridge Structural Database (CSD) [50]. The 3D structure of R-(−)-MIA·HCl was obtained after configuration inversion of the prepared S-(+)-MIA·HCl, followed by the structure optimization in the MM+ force field with the Polak–Ribière conjugate gradient algorithm (terminating at a gradient of 0.05 kcal mol^−1^ Å^−1^) using the Portable HyperChem 8.0.7 Release (Hypercube, Inc., Gainesville, FL, USA). The docking calculations were performed using a protonated form of MIA. The X-ray structures of cyclodextrins used for molecular modeling studies were taken from the CSD (The Cambridge Crystallographic Data Centre (CCDC) refcode: 648855 [38] for entries from Figure 5,Figure 6,Figure 7, ALIGAE [39] for entries A-D from Figure 8,Figure 9, BEFJOL [40] for entries A and C from Figure 10, and JOSWOD [41] for entry B and D from Figure 10). After the standard preparation procedures (including removal of water molecules and other ligands as well as an addition of hydrogen atoms and Gasteiger charges to each atom [51,52]), docking analysis was carried out with appropriate stoichiometry of cyclodextrin units.

Molecular Docking

Docking studies were carried out using Autodock Vina 1.1.2 software (The Molecular Graphic Laboratory, The Scripps Research Institute, La Jolla, CA, USA) [53] with exhaustiveness, num_modes, and energy_range parameters set as 8, 30, and 10, respectively. For the docking studies the corresponding grid box parameters were used:

- entry A and B from Figure 5 (three molecules of β-CD I-II-III): a grid box size of 20 Å × 20 Å × 20 Å centered on the C47 atom (x = −5.017, y = 1.413, z = 0.074);
- entry A and C from Figure 6 (two molecules of β-CD I-II): a grid box size of 20 Å × 20 Å × 20 Å centered on the C45 atom (x = 5.849, y = 3.007, z = −5.646);
- entry B and D from Figure 6 (two molecules of β-CD II-III): a grid box size of 20 Å × 20 Å × 20 Å centered on the C23 atom (x = 4.807, y = 1.076, z = 7.878);
- entry A and D from Figure 7 (one molecule of β-CD I): a grid box size of 20 Å × 20 Å × 20 Å centered on the C45 atom (x = 5.849, y = 3.007, z = −5.646);
- entry B and E from Figure 7 (one molecule of β-CD II): a grid box size of 20 Å × 20 Å × 20 Å centered on the C43 atom (x = 5.243, y = 0.841, z = 1.262);
- entry C and F from Figure 7 (one molecule of β-CD III): a grid box size of 20 Å × 20 Å × 20 Å centered on the C43 atom (x = 4.602, y = −1.221, z = 8.714);
- entry A and B from Figure 8 (two molecules of TMβ-CD I-II): a grid box size of 20 Å × 20 Å × 20 Å centered on the C11 atom (x = 3.352, y = 6.710, z = 2.402);
- entry A and C from Figure 9 (two molecules of TMβ-CD I): a grid box size of 20 Å × 20 Å × 20 Å centered on the C11 atom (x = 3.352, y = 6.710, z = 2.402);
- entry B and D from Figure 9 (one molecule of TMβ-CD II): a grid box size of 20 Å × 20 Å × 20 Å centered on the C10 atom (x = 6.775, y = 12.684, z = 10.278);
- entry A and C from Figure 10 (one molecules of DMβ-CD) a grid box size of 20 Å × 20 Å × 20 Å centered on the C39 atom (x = 17.161, y = 5.028, z = 4.351);
- entry B and D from Figure 10 (one molecule of RMβ-CD): a grid box size of 20 Å × 20 Å × 20 Å centered on the C45 atom (x = 2.967, y = 2.155, z = −4.366).

Graphic visualizations of the 3D model for the poses with the lowest free energies of binding were generated using VMD 1.9 software (University of Illinois at Urbana–Champaign, Urbana, IL, USA).

#### 3.2.6. Cell Culture and Chemical Treatment of Cells

The study is a continuation of the earlier work [10], and to keep the same experimental conditions, the Chinese hamster cells (B14 cell line) (purchased from Child Health Center in Warsaw (Poland)) were grown as a monolayer in Dulbecco’s Modified Eagle’s Medium (DMEM), supplemented with 10% fetal bovine serum. Located in 96-well microtiter plates were cells with an initial density of 1.5 × 10^5^ in 100 µL per well. The experimental conditions (rather high seeding density) have been chosen because, for less cells per well, the measured absorbance was too low to be meaningful for the assessment of changes, so we used a larger number of cells. Next, the 200 µM mianserin hydrochloride solution or heptakis (2,6-di-O-methyl)-β-cyclodextrin solutions from 0 to 1000 µM were placed with them at 310 K in a 5% carbon dioxide to 95% air atmosphere with more than 95% humidity for 24 and 48 h. The rest cells were treated with MIA+DM-β-CD solutions, with a changing molar ratio at the same conditions. The control cells and all the rest of the cells were recovered by gentle washing with PBS (pH = 7.4) twice. The cell viability was measured by 3-(4,5-dimethylthiazol-2-yl)-2,5-diphenyltetrazolium bromide (MTT) assay, performed according to Hansen et al. [54]. After 3 h of incubation, cells with 50 µL of MTT dissolved in PBS at 5 mg/mL, and the MTT-containing medium was removed. Next, DMSO (100 µL) was added to all wells [10]. The converted dye absorbance was measured at 570 nm by a microplate spectrophotometer (BioTek). Afterwards, the cell viability was calculated as the ratio of the sample absorbance in relation to the control absorbance, and expressed as a percentage and average ± S.D. The Student’s *t*-test was applied in evaluation of the statistical significance between the control and the treated groups, and *p* < 0.05 was accepted as statistically significant. The toxicity of the complexes was tested disregarding the 1:3 molar ratio, as the toxicity for the lower proportion appeared to be independent of it. Toxicity tests at the higher ratio of 1:4 were performed to support this assumption (Figure 12).

## 4. Conclusions

The mianserin hydrochloride with a heptakis (2,6-di-O-methyl)-β-cyclodextrin complexation process runs spontaneously with the release of thermal energy. The enthalpy and the entropy are favorable driving forces of MIA and DM-β-CD interactions, but the entropic term controls the ultimate stability of the complex. The binding constant of MIA inclusion inside the DM-β-CD cavity is higher than for the native β-CD. The stoichiometry coefficient value of the complex formation between DM-β-CD and MIA molecules obtained from ITC placed near to the ratio of 1.5. The ESI-MS spectrum pointed out the predominant presence of an MIA and DM-β-CD combination in the molecular ratio 1:1. This possibly indicates that connections with higher stoichiometry are less stable under the measurement conditions of this technique (ESI-MS), such as primarily high temperature, (593 K) while the ITC measurements were carried out at 298 K. The induced circular dichroism signals presented the strong negative Cotton effect, and an almost twofold weaker positive effect for the case of the MIA and DM-β-CD adduct. This phenomenon can be explained by the fact that the MIA molecule has at the same time the parallel and perpendicular orientation of the electric transition dipole moment of chromophores to the Z-axis of the DM-β-CD cavity, because it has four rings forming a shape similar to a bent letter T, with the chromophores almost perpendicular to each other. However, as some authors stated, the parallel-polarized electronic transition along the axis of the cavity should give an ICD signal that is two times larger with the opposite sign, as compared with the ICD signal caused by a perpendicular-polarized one. Presented results showed the reversed situation, where there were two times more intense negative ICD signals than positive ones. The explanation is very simple: the molecule of MIA with the letter T shape is inserted inside the DM-β-CD cavity probably more by the side of the D-ring (Figure 1), with other A-, B-, and C-rings suspended partially outside the DM-β-CD cavity, which is clearly visible in the results from the molecular docking presented in Figure 10. At the concentration 1000 µM of DM-β-CD, the viability of B14 cells decreased to 53% in relation to the control, and the viability of the cells incubated with complexes MIA+DM-β-CD decreased even more, to about 6–7% in relation to the control. The very toxic effect of MIA+DM-β-CD is rather the same for all complexes used in the molar ratio from 1:1 to 1:4. The increase in toxicity of MIA in the presence of DM-β-CD is in opposition to the protective effect of β-CD complexed with MIA. It can be assumed that in contrast to β-cyclodextrin molecules, heptakis (2,6-di-O-methyl)-β-cyclodextrin molecules do not form conglomerates of more than one host molecule so easily, and therefore do not have the ability to reduce the cytotoxicity of the included drug. Furthermore, this is next to the toxic properties of the DM-β-CD molecules alone. Thus, the methylation in the two and six position of β-CD changed the biological properties of MIA in the β-cyclodextrin complexes. Moreover, another possible reason for the higher toxicity of the complex with mianserin could be the increased permeation of the drug, as a result of the cholesterol sequestration from the plasma membrane by the cyclodextrin [55,56,57]. This toxicity gain under the influence of DM-β-CD molecules may be used for substances with anti-tumor cell activity, but in the case of MIA, it is not recommended to use it with DM-β-CD as an antidepressant drug only. It is possible, however, that the connection of mianserin hydrochloride with heptakis (2,6-di-O-methyl)-β-cyclodextrin has some anti-tumor cell potential, or in Leishmaniasis treatment.

## Figures and Tables

**Table 1:** Stoichiometry coefficients of binding (n), binding constants (K), enthalpy (ΔH), entropic effects (TΔS), and the Gibbs free energies values (ΔG) obtained for the complex formation of mianserin hydrochloride with β-cyclodextrin or heptakis (2,6-di-O-methyl)-β-cyclodextrin at T = 298.15 K under atmospheric pressure *p* = 101 kPa.

	n	K/M^−1^	ΔH/kJ·mol^−1^	TΔS/kJ·mol^−1^	ΔG/kJ·mol^−1^
MIA					
β-CD *	2.15	1320	−3.24	14.55	−17.79
DM-β-CD **	1.64 ± 0.15	1690 ± 210	−4.72 ± 0.09	13.70 ± 0.99	−18.42 ± 0.28
SRT					
β-CD ^#^	1.20	5820	−20.44	1.06	−21.53
DM-β-CD ^#^	1.60	7960	−14.20	7.96	−22.19

* Reference [14]. ** This work. The uncertainties are standard deviation of an average value from four independent measurements. **^#^** Reference [22].

**Table 2:** The values of free energies of binding for MIA enantiomers and chosen β-CD molecules obtained from molecular docking results.

Representative Geometry	Crystal Structure Refcode from CSD and Amount of Receptor Molecules	S-(+)-MIA·HCl	R-(−)-MIA·HCl
		Free Energies of Binding kcal∙mol^−1^ (kJ∙mol^−1^)	
A and B Figure 5	648855 (three molecules of β-CD I-II-III)	−7.5 (−31)	−8.5 (−35)
A and C Figure 6	648855 (two molecules of β-CD I-II)	−7.1 (−30)	−8.2 (−34)
B and D Figure 6	648855 (two molecules of β-CD II-III)	−6.6 (−28)	−7.6 (−32)
A and D Figure 7	648855 (one molecule of β-CD I)	−6.1 (−26)	−6.2 (−26)
B and E Figure 7	648855 (one molecule of β-CD II)	−5.9 (−25)	−6.1 (−26)
C and F Figure 7	648855 (one molecule of β-CD III)	−6.2 (−26)	−6.2 (−26)
A and B Figure 8	ALIGAE (two molecules of TMβ-CD I-II)	−8.0 (−33)	−8.0 (−33)
A and C Figure 9	ALIGAE (one molecule of TMβ-CD I)	−6.5 (−27)	−6.4 (−27)
B and D Figure 9	ALIGAE (one molecule of TMβ-CD II)	−6.2 (−26)	−6.6 (−28)
A and C Figure 10	BEFJOL (one molecule of DMβ-CD)	−6.4 (−27)	−6.8 (−28)
B and D Figure 10	JOSWOD (one molecule of RMβ-CD)	−6.8 (−28)	−6.8 (−28)

**Table 3:** Sample Table. ^1^

Name	Alternative or IUPAC Name	CAS Number	Source	Molecular Weight g∙mol^−1^	Mass Fraction Purity as Stated by Supplier
mianserin hydrochloride	(1, 2, 3, 4, 10, 14b-hexahydro-2-methyldibenzo [c, f]pyrazino [1, 2-a]azepine hydrochloride	21535-47-7	Sigma-Aldrich	300.83	0.98
heptakis(2,6-di-O-methyl)-β-cyclodextrin	2,6-Di-O-methyl-β-cyclodextrin	51166-71-3	CycloLab	1331.36	0.95
MTT	3-(4, 5-Dimethyl-2-thiazolyl)-2, 5-diphenyl-2H-tetrazolium bromide	298-93-1	Sigma-Aldrich	414.32	0.98

^1^ The substances were dried under reduced pressure at T = 298 K for 72 h.

## Data Availability

Not applicable.
